# The variability and predictors of quality of AIDS care services in Brazil

**DOI:** 10.1186/1472-6963-9-51

**Published:** 2009-03-20

**Authors:** Maria Ines Battistella Nemes, Regina Melchior, Cáritas Relva Basso, Elen Rose Lodeiro Castanheira, Maria Teresa Seabra Soares de Britto e Alves, Shaun Conway

**Affiliations:** 1 Department of Preventive Medicine, Faculty of Medicine, University of Sao Paulo, Sao Paulo, Brazil; 2 Department of Public Health, State University of Londrina, Paraná, Brazil; 3 Department of Public Health, Faculty of Medicine of Botucatu, UNESP, São Paulo State University, Brazil; 4 Department of Public Health, Federal University of Maranhão, Maranhão, Brazil; 5 London School of Hygiene and Tropical Medicine Health Policy Unit, London, UK

## Abstract

**Background:**

Since establishing universal free access to antiretroviral therapy in 1996, the Brazilian Health System has increased the number of centers providing HIV/AIDS outpatient care from 33 to 540. There had been no formal monitoring of the quality of these services until a survey of 336 AIDS health centers across 7 Brazilian states was undertaken in 2002. Managers of the services were asked to assess their clinics according to parameters of service inputs and service delivery processes. This report analyzes the survey results and identifies predictors of the overall quality of service delivery.

**Methods:**

The survey involved completion of a multiple-choice questionnaire comprising 107 parameters of service inputs and processes of delivering care, with responses assessed according to their likely impact on service quality using a 3-point scale. K-means clustering was used to group these services according to their scored responses. Logistic regression analysis was performed to identify predictors of high service quality.

**Results:**

The questionnaire was completed by 95.8% (322) of the managers of the sites surveyed. Most sites scored about 50% of the benchmark expectation. K-means clustering analysis identified four quality levels within which services could be grouped: 76 services (24%) were classed as level 1 (best), 53 (16%) as level 2 (medium), 113 (35%) as level 3 (poor), and 80 (25%) as level 4 (very poor). Parameters of service delivery processes were more important than those relating to service inputs for determining the quality classification. Predictors of quality services included larger care sites, specialization for HIV/AIDS, and location within large municipalities.

**Conclusion:**

The survey demonstrated highly variable levels of HIV/AIDS service quality across the sites. Many sites were found to have deficiencies in the processes of service delivery processes that could benefit from quality improvement initiatives. These findings could have implications for how HIV/AIDS services are planned in Brazil to achieve quality standards, such as for where service sites should be located, their size and staffing requirements. A set of service delivery indicators has been identified that could be used for routine monitoring of HIV/AIDS service delivery for HIV/AIDS in Brazil (and potentially in other similar settings).

## Background

AIDS is a complex chronic condition and affected patients require well-functioning, comprehensive healthcare services [1]. Early studies have found that the quality of AIDS care provided varies considerably across different settings [2,3]. We know that the quality of care is affected by the availability of service inputs and by service delivery processes [4,5]. Although many of these factors have not yet been shown to be correlated with treatment outcomes, most reflect generally accepted standards for care delivery [6-8]. In addition, there is evidence to suggest that some of the key organizational characteristics of primary care, such as accessibility and comprehensiveness of services, are related to HIV patient outcomes [9].

The Brazilian policy of universal and free access to HIV/AIDS care and treatment has led to a marked increase in the number of sites providing AIDS care, from 33 in 1996 to 540 in 2002. These sites are located in primary care units, general or sexually transmitted diseases/AIDS dedicated clinics, and hospital outpatient departments.

All care sites are expected to deliver antiretroviral therapy in accordance with the Brazilian National Consensus on Antiretroviral Therapy. The central National STD and AIDS Program supplies antiretroviral medicines and the laboratory network for CD4, viral load and genotype tests. In addition, the national program makes general recommendations for implementing services and delivering HIV/AIDS care, such as the need to have a multidisciplinary team (including at least clinicians, nurses and psychologists) to provide voluntary counseling HIV testing and support to treatment adherence. Other service inputs and delivery arrangements are determined according to local circumstances and health service organization at the decentralized level.

The national program has not made recommendations regarding quality standards and there is no monitoring of sites to ensure quality of care. Prior to our survey in 2002 (the results of which have been published elsewhere [10], there had been no external assessment of how HIV/AIDS care services are structured or organized. This study provides further analysis of the data from this cross-sectional survey of HIV/AIDS care services at 336 sites in 7 Brazilian states, assesses the degree to which service parameters vary among sites, and determines whether these parameters could be valid indicators of service quality for routine monitoring.

## Methods

The survey was conducted at all sites that were identified as delivering antiretroviral treatment in 7 of the 27 Brazilian states selected by the Brazilian STD/AIDS Program Coordination Office, see table 1.

**Table 1:** Number of HIV/AIDS health services and patients under ART according to Brazilian State

***State*** ***(Region)***	***Number of AIDS health services***	***Number of patients under ART***
Sao Paulo (Southeast)	171	49655
Rio de Janeiro (Southeast)	96	23293
Rio Grande do Sul (Southem)	45	14297
Mato Grosso do Sul (Midwest)	12	1042
Pará (North)	5	1330
Ceará (Northeast)	3	1952
Maranhão (Northeast)	4	831
**Total in the study**	**336**	**92,400**

**Total in Brazil**	**540**	**128,870**

This survey investigated parameters relating to health service inputs (infrastructure and resources) and service delivery processes (organizational and managerial factors) as proxy indicators of the quality of these services [11]. These parameters had previously been selected by a detailed analysis of five AIDS care services [12,13] involving focus-group discussions with patients and doctors [14] and consultations with program managers from the national and state-level program coordinating offices. We also reviewed the literature on quality of HIV/AIDS care and national guidelines on ART [15].

The self-administered questionnaire comprised 157 multiple-choice questions. Two six-member panels of experts (one representing providers and the other service managers) assessed the likely reliability, validity, and feasibility of the instrument. The survey was piloted and reviewed by 31 randomly selected health services managers not included in the survey sample. As a result of these procedures, 42 questions were eliminated as they were not considered useful. The final version assessed 107 parameters: 30 were related to service inputs and 77 to service delivery processes, of which 47 were relevant to the organization and 30 to the management of service delivery, see table 2. Only objectively quantifiable measures were used for service managers to assess their services. See additional file 1 – Questionnaire English Short Version and additional file 2 – Questionnaire Portuguese Full Version.

**Table 2:** Overview of the mean parameters investigated in the study

**SERVICE INPUTS**	**ORGANIZATION**	**MANAGEMENT**
▪ doctors experienced in providing HIV care	▪ pre-booked appointments	▪ manager's professional profile
▪ staffing ratios for nurses, social workers, psychologists, dentists, pharmacists	▪ follow-up appointment booking	▪ manager's responsibilities
▪ auxiliary personnel	▪ length of booking interval	▪ patient registration and record-keeping
▪ availability of medical specialties for referral	▪ length of consultation	▪ data security and patient confidentiality
▪ medication supplies (ARV and others)	▪ caseload	▪ confidential reminder system for non-attendants
▪ radiology services and laboratory tests	▪ non pre-booked appointments	▪ regular team meetings
▪ occupational bio-safety	▪ referrals among professionals	▪ planning and monitoring processes
▪ public transportation to access service	▪ waiting time in the waiting room	▪ staff training
▪ hours of operation and working days	▪ counseling at the time of HIV test	▪ community linkages
▪ physical accessibility	▪ counseling on safer sex and family planning	
	▪ use of guidelines and written protocols	
	▪ activities to support treatment adherence	

Standardized responses were provided using a three-point scale that represented performance levels against expected service standards. Achievement of the "minimum expected standard" was assigned a score of 1, performance above the minimum standard scored 2, and performance below the standard scored 0, see table 3.

**Table 3:** Examples of point scale

**Dimension**	**Indicator**	**Scale**
INPUTS	One part time doctor for more than 200 patients	0
	One part time doctor for 151 to 200	1
	One doctor for 150 patients or fewer.	2

ORGANIZATION OF SERVICE DELIVERY	Gynecological medical consultation not available	0
	Gynecological medical consultation for patients with symptoms reported or requesting referral	1
	Routinely, noncompulsory offering to all female patients	2

MANAGEMENT OF SERVICE DELIVERY	No regular team meetings	0
	Regular team meetings with part of the team	1
	Regular team meetings for all members of the team (including doctors)	2

The questionnaire was completed at 27 service sites that had previously been qualitatively assessed and stratified according to their levels of service quality [16]. To avoid the central tendency measurement effect for stratifying service levels, we used K-means clustering, which uses Euclidian distances to determine the center of the possible groups [17]. This allowed us to compare how well the ratings performed through these two forms of assessment. Overall, there was a good degree of correspondence between the assessment methods (no center with poor qualitative evaluation had a high score in the K-means comparison or vice versa). The scoring exercise was also performed at 12 centers in 3 states (4 each) not included in the survey sample that were classified according to care quality by state program managers. There was agreement between the manager's opinion and the score obtained using the questionnaire. These procedures were interpreted as validation of the questionnaire.

To improve the response rate, the objectives of the survey were communicated by visits to program regional officers and national and regional HIV program meetings.

The survey was sent to the managers of the 336 selected sites, and reminders were sent after 3 and 6 weeks.

Responses were grouped into groups of likely service quality according to the K-means analysis. To analyze the associations between institutional characteristics and the service quality, the dependent variable was defined as inclusion in the better service quality groups.

The health service institutional characteristics that we considered potential predictors of better service quality were: state (São Paulo/Rio de Janeiro/others), number of inhabitants in the municipality (>400,000/<400,000), type of service (exclusively for STD/AIDS or not), length of time the center has provided HIV/AIDS care (>5 years/<5 years), and number of patients (>100/101–500/>500). Missing variables were not considered in the denominator.

Pearson's chi-squared test was used to estimate differences in each variable among the groups. Odds ratios and 95% confidence intervals were estimated for each independent variable category, considering one of the categories as the comparison for the others compared with the other categories.

A stepwise retrospective logistic model was used to estimate the independent effect of the variables. Variables with p-values < 0.2 were adjusted in a full model. Those with p > 0.10 were removed step by step. The importance of removed variables for the model was evaluated using the likelihood ratio test. The analysis was performed using SPSS 8.0 and S-Plus 4.5 software

The Sao Paulo University School of Medicine Ethics Committee approved the study. Health service managers signed voluntary informed consent and were assured that the results for their site would not be identifiable in any reports and that the assessment would have no effect on them from the government program.

## Results

The questionnaire was answered by 95.8% (322) of managers of health services surveyed, see table 4.

**Table 4:** Institutional Characteristics of Health Services Surveyed*.

**Characteristic**	**No (%)**
**State**	
Ceará	3 (1.0)
Maranhão	4 (1.2)
Pará	5 (1.5)
Mato Grosso do Sul	12 (3.7)
Rio Grande do Sul	34 (10.6)
Rio de Janeiro	94 (29.2)
São Paulo	170 (52.8)
No of inhabitants of the municipality**	
> 400,000 inhabitants	119 (36.96)
< 400,000 inhabitants	203 (66.04)
**N° of patients**	
> 500	58 (18.0)
101–500	107 (33.2)
< 100	157 (48.8)
**Type of service**	
Non-exclusive	278 (86.34)
Exclusively HIV/AIDS and STD	42 (13.0)
**Duration of delivering services**	
> 5 years	210 (65.2)
≤ 5 years	112 (34.78)

* Health service managers from eleven services from state of Rio Grande do Sul, two from Rio de Janeiro, and one from Sao Paulo failed to respond. The average of institutional characteristics of non-respondents from Rio Grande do Sul did not differ from the respondents.

** Source: The Brazilian Institute of Geographic and Statistics (IBGE)

The overall mean quality score was 1.128 (56% of our expected benchmark). The lowest mean score was 0.563 and the highest was 1.680.

The overall mean score for service input parameters was 1.189. The highest individual mean score was for the availability of antiretroviral drugs (1.134), whereas the lowest mean score related to the availability of medicines for opportunistic infections (0.457).

The overall mean score for service delivery process parameters was 1.117. The care organization component of this had a mean value of 1.139. Services received the highest scores for pre booked consultations for patients (1.882) and lowest scores for non pre-booked patient care patient care routines (0.215). Service management activities had an overall mean of 1.036, with the highest mean scores related to epidemiological data registration (1.749) and the lowest to monitoring activities (0.635).

Variability was greater for process (11.38 to 288.96%) than for service inputs (26.47 to 195.10%)

**Figure 1:**
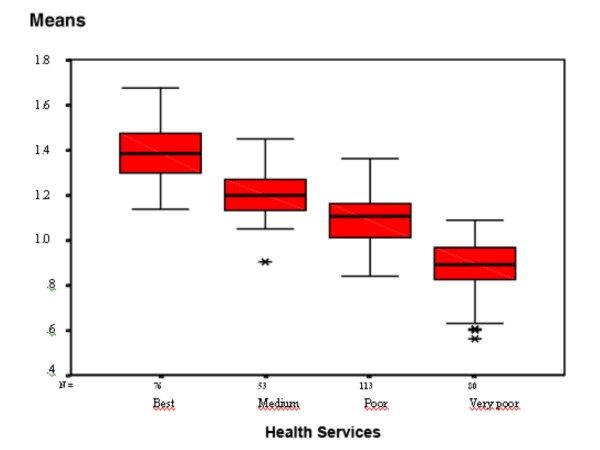
**Health services grouped according to K-means clusters of scores**.

Analysis of the sum of K-mean residual square revealed four service levels: 76 services (24%) were scored as quality level 1 (best), 53 (16%) as level 2 (medium), 113 (35%) as level 3 (poor), and 80 (25%) as level 4 (very poor), see figure 1. ANOVA was used to confirm differences between group arithmetic means.

Parameters relating to service delivery processes had the greatest effect on which quality group each service site would be classified. With a cut-off point for differences between means ≥ 0.25, 91% (70) of process parameters were associated with at least two of the groups, compared with 83% (25) of the service input. In all groups, the process parameters had higher proportion of zero scores compared with the service input parameters.

No statistically significant associations were found between better quality service (best and medium groups) and the geographical state or number of years since the service was established. The number of inhabitants within a municipality, the number of patients, and type of service were all included in the final logistic model, see table 5.

**Table 5:** Logistic model for inclusion in the better quality groups ("best" and "medium") according to health service characteristics.

**Characteristics**	OR (95% CI)*	OR (95% CI)**	**P > |z|**	LR (p)***
**Type of municipality**				176 (0.0003)
<400,000 inhabitants	1.0	1.0		
>400,000 inhabitants	5.3 (3.2–8.7)	3.0 (1.7–5.4)	0.000	

**No. of patients**				177 (0.0005)
<100	1.0	1.0		
101–500	5.0 (2.9–8.7)	3.0 (1.6–5.4)	0.001	
>500	8.3 (4.2–16.3)	3.4 (1.5–7.7)	0.002	

**Type of service**				181 (0.0000)
Not exclusive	1.0	1.0		
Exclusive (HIV/AIDS and STD)	9.8 (4.2–22.9)	7.5 (3.0–19.0)	0.000	

*Crude odds ratio

** adjusted odds ratio

*** likelihood ratio

## Discussion

### Overall quality of services

Most surveyed sites scored approximately 50% of our benchmark expectation. Overall, service inputs parameters scored higher and were more homogeneous than those assessing the processes of service delivery. Nevertheless, some sites had low scores for service inputs parameters mostly related to lack of medical specialist services and medicines for opportunistic infections. These inputs are dependent on the infrastructure of the local healthcare system. However, those service inputs directly provided by the federal level of the system (antiretrovirals and essential laboratory tests) showed an overall better availability.

The size and composition of clinical care teams showed marked variability among the sites. Some sites (21%) have only a single clinician; this has previously been associated with deficiencies in HIV patient care [18].

Many sites had low scores for parameters relating to service delivery processes, including some sites that had high scores for service inputs. Many of these process parameters are considered crucial for good quality AIDS care, such as 'identifying groups at risk of non-adherence [19,20]. For instance, many of these sites were not organized to appropriately deal with patients who miss appointments and who are at greater risk of non-adherence [21]. Most services seemed to focus only on patients' immediate care needs while failing to address the reasons for missed appointments or to re-engage patients in the follow up process.

Parameters related to care delivery organization are strongly dependent on the attitude of and processes developed by the service manager. The lowest overall scores were for parameters relating to service management. For example, the patient record systems at many sites were unable to provide even basic service monitoring information such as the number of consultations per patient and record of investigations undergone by each patient. Missed appointment rates (a feasible and reliable indicator of poor adherence) were routinely tracked by only 25% of sites. In 53% of sites, no regular technical/professional team meetings were held.

The results highlight the need for greater standardization and fairness in the distribution of resources across service sites and promotion of continuous improvement through training and to strengthen management capacity.

### Predictors of quality of services

This study has shown that some institutional characteristics of the services are associated with input and process parameters of the quality of care provided. Some of these associations are unsurprising, such as the finding that specialist services are more likely to be available in large municipalities. These associations are useful to highlight variations in service characteristics that are associated with underlying structural problems, such as urban-rural differences in the distribution of healthcare resources and the historical development of national program HIV-related services (with most of the large, better-resourced, specialized services located in the major cities where the HIV epidemic began).

Early studies from other regions (particularly North America) have associated better-quality HIV services with delivery via specialized clinics and by more experienced clinical providers [22-26]. This also seems valid in Brazil because the presence of specialized services at a site was independently associated with higher service quality scores in the overall assessment. There was no similar association with the time since establishment of a service, indicating that several older centers could have serious service deficiencies.

Large numbers of patients were also predictive of better service quality. Other studies have previously suggested a positive association between large numbers of patients and better quality HIV care [27-29]. Although site size is a controversial proxy of quality of care for general practices [30,31], it seems to apply to complex conditions that require a wide range of laboratory and specialist inputs such as AIDS. As we have seen in our qualitative studies, larger practices are better able to find resources and generate the case load needed to support specialist provision and multidisciplinary working. We have performed a retrospective study at the same services and the results support our concerns about small care sites, because we found that services with fewer than 100 patients had high non-adherence rates [21].

### Implications for Brazilian AIDS care policy

Our findings indicate that the current distribution of Brazilian AIDS healthcare services needs to be reviewed. In the North, Northeast, and Midwest states (Pará, Ceará, Maranhão, and Mato Grosso do Sul) where HIV prevalence is low [32], the number of available services seems inadequate to ensure adequate access to care, particularly owing to the large sizes of these states. To improve access in these regions, primary care sites should temporarily be responsible for HIV patients. However strong cooperation is needed between the few specialized services and primary care sites to deliver services of acceptable quality; this is particularly important to increase the expertise of primary care generalist physicians [33,34].

In the Southeast and Southern regions (Rio Grande do Sul, Rio de Janeiro, Sao Paulo), the AIDS epidemic has been much more severe [32]; however, these regions are much smaller and transport infrastructure is much better compared with the North, Northeast and Midwest states. Therefore, there seems to be little justification for the 99 services (36% of the total in these regions) that treat fewer than 50 patients.

In the decentralized Brazilian health system, commissioning HIV treatment sites is largely the responsibility of municipal government. However, the central level of the AIDS program does have political legitimacy and resources to affect such decisions once it has responsibility for essential inputs such as availability of antiretroviral therapy and laboratory tests. In addition, a national technical standard involving more rigorous assessment of the local situation and need for services could improve the quality of services.

### Limitations and strengths of the study

The main limitation of this study is that it only focused on parameters relating to service inputs and service delivery processes to assess the quality of HIV care services. The study design did not allow for direct validation, such as by assessing outcomes. At the time of this survey, it was not possible to record the source of clinical outcome data, other than by directly reviewing medical records at a large number of service sites, which was not feasible in this study.

However, a number of structure and process indicators have historically been used as indicators of the quality of healthcare [8,35,36]. In the present study, we tried to maximize the validity of the assessment by a systematic method of development and application of the parameters investigated [37]. We also assessed the validity of these parameters as indicators of service quality by comparing them with ratings obtained from qualitative assessments at a subgroup of sites.

Another possible limitation is the response bias of service managers completing the survey, although we believe that this was minimized by effectively communicating the purpose and confidentiality of the survey. In addition, response options were based on objectively verifiable measures.

This was largely a descriptive study. The logistic regression analysis was used to highlight the effects of a small set of service characteristics associated with better service quality. Although these characteristics are far from explaining differences in service quality, we believe they are useful to give AIDS program decision-makers an overview of the distribution of AIDS care services in Brazil.

Despite its limitations, we therefore believe that the parameters identified in the present study are useful for establishing a service profiles as a constructive first step in formally evaluating and monitoring the quality of HIV care in Brazil.

The summary results of this study have been provided to all participating services, together with their individual site assessment scores and quality rating. The study data have been provided to national and regional program coordination offices. We have also presented the survey results at both national and regional program offices, as well as at many scientific and technical HIV meetings.

In 2005, we adapted the questionnaire into an electronic self-administered tool to allow service managers to evaluate their own services and compare their scores, as well as to provide data to regional and national programs. Good practice recommendations have been developed for each indicator and these are now available as online resources [38].

Prior to 2008, the Brazilian National STD and AIDS program had not formally introduced routine monitoring and evaluation of the quality of HIV/AIDS services. However, the electronic survey tool that we have developed has since been recommended for use by all service sites for annual service monitoring processes, and the first results are due at the end of 2008. This will be a good opportunity to compare the scores with those from our original survey 6 years ago and to re-examine the appropriateness of the indicators.

## Conclusion

This study is the first comprehensive assessment of service inputs and delivery processes of HIV/AIDS healthcare services in Brazil. Parameters relating to service inputs and the service delivery processes were used to indicate the quality of HIV/AIDS care being delivered. Findings indicate that the quality of services is highly variable across the country.

Service input parameters tended to be consistently associated with higher scores compared with service delivery process parameters. This indicates that there is a need to strengthen local service management capacity.

Non-specialized services and those that treat fewer than 100 patients were more likely to have low overall quality scores. This is a particular challenge for the Brazilian National STD and AIDS Program given the relatively high proportion of non-specialized and small service sites in many states, and the distribution of AIDS care services may need to be reviewed at some locations. More sites are needed at some locations to ensure access, whereas at other locations it might be better to keep only specialized larger practices.

The Brazilian AIDS response has been recognized for achieving many good results. However, it is necessary to improve the service quality and to ensure that this is achieved equitably throughout Brazil. The lack of monitoring of service delivery standards could have reduced the effectiveness of the services, and the small and non-specialized care sites might have been more adversely affected. Further research is needed to confirm the results of this study, as well as to investigate how clinical outcomes are affected by the studied health service parameters.

## Competing interests

The authors declare that they have no competing interests.

## Authors' contributions

MIBN coordinated the conception, design, acquisition, analysis and interpretation of data and, writing of the final manuscript. RM and CRB contributed to design, acquisition, analysis and interpretation of data and have been involved in drafting the manuscript. ERLC and MTSSBA contributed to design, analysis and interpretation of data and have been involved in drafting the manuscript. SC contributed to analysis and interpretation of data and has been involved in drafting the manuscript.

All authors read and approved the final manuscript.

## Pre-publication history

The pre-publication history for this paper can be accessed here:



## Supplementary Material

Additional file 1 **English version of 15 the multiple choice questions included in the questionnaire used in the survey**. Click here for file

Additional file 2 **Full Portuguese version of the questionnaire with 157 questions used in the survey**. Click here for file
