# Supplementary material for: The variability and predictors of quality of AIDS care services in Brazil
Source: BMC Health Serv Res. 2009 Mar 20;9:51. doi: 10.1186/1472-6963-9-51 (PMC2671500; doi:10.1186/1472-6963-9-51)
Supplement: Additional file 1 — English version of 15 the multiple choice questions included in the questionnaire used in the survey. [file 1472-6963-9-51-S1.doc]

**
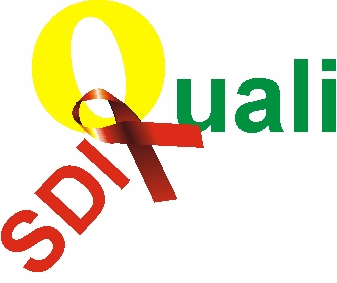
**

**QUALIAIDS QUESTIONNAIRE**

**SHORT VERSION**

**QUALITY OF CARE SELF- ASSESSMENT TOOL TO HEALTH SERVICES PROVIDING OUTPATIENT CARE TO PEOPLE LIVING WITH AIDS.**

**This is the *Qualiaids* questionnaire short version for English speakers. Fifteen questions were translated into English, in order to give some examples of this instrument for a broader range of people.**

q1 – How many HIV**/**AIDS patients are currently been followed in the health service?

**(Only one answer allowed)**

( ) Number of patients older than 13 years old

( ) It is not possible to obtain this data

q2 – How many days a week does the health service offer HIV/AIDS treatment and care?

**(Only one answer allowed)**

(1) Once a week

(2) Twice a week

(3) 3 days a week

(4) 4 days a week

(5) 5 days a week

(6) More than 5 days a week

q3- Describe how many professionals are in charge of ambulatory care to STI/HIV/AIDS patients in the health service.

| Professionals | Total number of professionals | Number of health workers according to  partial or full time availability | |
| --- | --- | --- | --- |
|  | (A) - | (B)  Full time | (C) Part time |
| (1) Nurse |  |  |  |
| (2) Psychologist |  |  |  |
| (3) Dentist |  |  |  |
| (4) Pharmacist |  |  |  |
| (5) Social Worker |  |  |  |
| (6) Assistant nursing |  |  |  |

q4 – Within the health service, indicate which of the following information are routinely recorded.

***Caution: Items 1 to 9 are related to procedures and 10 to 18 to patients***

1. Number of procedures and medical consultations

(2) Number of medical consultations per age

(3) Number of medical consultations according to the co-morbidity (e.g. OI and other STI)
(4) Number of medical consultations per physician

(5) Number of group appointments

(6) Number of medical consultations for persons with occupational HIV exposure.

(7) Number of missed medical consultations.

(8) Number of missed medical consultations per patient

(9) Number of lab tests performed

(10) Number of patients per age

(11) Number of patients per sex

(12) Number of HIV+ patients

(13) Number of tuberculosis co-infected patients

(14) Number of patients per diagnosis (opportunist infections and other STI)

(15) Number of patients under ART

(16) Number of medical consultations per patient per year

(17) Number of deaths reported

(18) Number of patients per type of anti-retroviral regimen

(19) Other information

.

q5 – In case of a first time tested HIV+ person does not return to the health service, the health team

**(Only one answer allowed)**

1. Waits for a spontaneous return of the person
2. Calls confidentially the person
3. Calls confidentially the person only in case the person is a pregnant woman
4. This question does not apply, because HIV testing is anonymous
5. This question does not apply, because the health service only follow HIV+ patients and does not offer HIV testing.
6. This question does not apply, because the health service performs rapid HIV test

q6 – What is the average interval of time between the first contact of a HIV+ person with the health service and the first HIV/AIDS medical consultation (excluding the emergency cases)?

**(Only one answer allowed)**

(1) First medical consultation always occurs in the first contact with the service.

(2) Less than a week.

(3) From 1 to 4 weeks

(4) From 5 to 8 weeks

(5) More than 8 weeks.

q7 – Routine medical consultations are scheduled as:

**(Only one answer allowed)**

(1) Pre-booked appointment for each patient

(2) Pre-booked appointments for more than one patient in an hour, seen by the doctor according to their arrival.

(3) Pre-booked appointments for many patients in a period’s time (morning or afternoon), seen by the doctor according to their arrival.

(4) Each doctor has his/her own routine.

(5) Medical consultations are not pre-booked.

q8 – Which are the routines for accessing gynecological medical consultation in the health service?

(1) Patients actively choose to access (i.e., an "opt-in" approach)

(2) Routinely, noncompulsory offering to all female patients (i.e., an "opt-out" approach)

(3) Patients with gynecological symptoms reported

(4) There are no routines. Accesses depend on the case.

(5) The service does not have a gynecologist physician.

q9 – What is the average duration of medical consultations for patients starting ART?

**(Only one answer allowed)**

(1) 15 minutes

(2) 30 minutes

(3) 45 minutes

(4) 60 minutes

q10 – In relation to the desire of people living with HIV/AIDS (men and women) to have children, the health team:

(1) Does not recommend the conception due to the risks for the woman’s health.

(2) Does not recommend the conception due to the risk of mother to child transmission

(3) Advises the risks and suggests the best moment for conception.

(4) Does not recommend conception, but in case of pregnancy, provides appropriated care.

q11 – Check on the table below the anti-retroviral drugs available in the last six months

***Caution: this question is only applicable to settings where anti-retrovirals are purchased only by the institution providing treatment (governmental or NGO)***

| **Anti-retroviral drugs** | Not used; Not available* | Always available | Not available: stock-out in the last 6 months | | | |
| --- | --- | --- | --- | --- | --- | --- |
|  |  |  | **Up to 7 days** | **8 to 15 days** | **16 to 30 days** | **More than 30 days** |
| **Abacavir (ABV) tablets 300mg** |  |  |  |  |  |  |
| **Amprenavir (AMP) tablets 150mg** |  |  |  |  |  |  |
| **Atazanavir (ATZ) tablets 150mg** |  |  |  |  |  |  |
| **Atazanavir (ATZ) tablets 200mg** |  |  |  |  |  |  |
| **Didanosine (ddI) EC tablets 250mg** |  |  |  |  |  |  |
| **Didanosine (ddI) EC tablets 400mg** |  |  |  |  |  |  |
| **Didanosine (ddI) tablets 100mg** |  |  |  |  |  |  |
| **Didanosine (ddI) tablets 25mg** |  |  |  |  |  |  |
| **Efavirenz (EFV) tablets 200mg** |  |  |  |  |  |  |
| **Enfuvirtide (T20) 90 mg IV** |  |  |  |  |  |  |
| **Indinavir (INV) tablets 400mg** |  |  |  |  |  |  |
| **Lamivudine (3TC) tablets 150mg** |  |  |  |  |  |  |
| **Lopinavir/r (LPV) tablets 133mg** |  |  |  |  |  |  |
| **Nelfinavir (NFV) tablets 250mg** |  |  |  |  |  |  |
| **Nevirapine (NPV) tablets 200mg** |  |  |  |  |  |  |
| **Ritonavir (RTV) tablets 100mg** |  |  |  |  |  |  |
| **Saquinavir (SQV) hard tablets 200mg** |  |  |  |  |  |  |
| **Saquinavir (SQV) soft gel tablets 200mg** |  |  |  |  |  |  |
| **Stavudine (D4T) tablets 30 mg** |  |  |  |  |  |  |
| **Stavudine (D4T) tablets 40mg** |  |  |  |  |  |  |
| **Tenofovir (TFV) tablets 300mg** |  |  |  |  |  |  |
| **Zidovudine (AZT) IV** |  |  |  |  |  |  |
| **Zidovudine (AZT) tablets 100mg** |  |  |  |  |  |  |
| **Zidovudine (AZT) tablets 300mg** |  |  |  |  |  |  |
| **Zidovudine 300mg + Lamivudine 150mg** |  |  |  |  |  |  |

****Not available: not standardized by the current guidelines or not purchased for at least 6 months***

***Not used: there is no need for those drugs in reason of clinical and immunological patient’s profiles***

q12 - The availability of CD_4_ tests is:

**(Only one answer allowed)**

(1) 1 test per patient per year
(2) 2 tests per patient per year

(3) 3 tests per patient per year

(4) More than 3 tests per patient per year

(5) CD_4_ tests are not available

q13 – Does the service often have regular meetings for case studies and clinical procedures discussions?

**(Only one answer allowed)**

(1) No

(2) Yes, weekly

(3) Yes, every 15 days

(4) Yes, monthly

(5) Yes, only when it’s necessary

q14 – Which are the managerial specializations of the health service’s manager?

(1) The manager does not have such specializations

(2) Public health specialization

(3) Health management specialization

(4) Short training managerial courses.

(5) Others.

q15 – Did the service perform its annual activities planning in the last year by:

**(Only one answer allowed)**

(1) The manager, with the health team and the regional authorities.

(2) The manager, with the regional authorities.

(3) The regional authorities, without the manager and health team participation.

(4) The manager, the health team, the patient’s representatives, and regional authorities.
