# Supplementary material for: The variability and predictors of quality of AIDS care services in Brazil
Source: BMC Health Serv Res. 2009 Mar 20;9:51. doi: 10.1186/1472-6963-9-51 (PMC2671500; doi:10.1186/1472-6963-9-51)
Supplement: Additional file 2 — Full Portuguese version of the questionnaire with 157 questions used in the survey. [file 1472-6963-9-51-S2.pdf]

Ministério da Saúde
Secretaria de Vigilância em Saúde
Programa Nacional de DST/AIDS

Universidade de São Paulo
Faculdade de Medicina
Departamento de Medicina Preventiva
Equipe QualiAids

AVALIAÇÃO DA QUALIDADE DA ASSISTÊNCIA AMBULATORIAL NOS SERVIÇOS PÚBLICOS DE ATENÇÃO À AIDS NO BRASIL

Serviço: ____________________

Município/ Estado: ____________________

Endereço: ____________________

Responsável pelo serviço de HIV/AIDS: ____________________

Responsável pelo preenchimento do questionário: ____________________

Função: ____________________

Contatos para conferência: Telefone: __________ Fax: __________

E-mail: ____________________

I. Identificação e características gerais do serviço de HIV/Aids

1. Qual a instituição **gestora**** deste serviço?

Assinale apenas uma alternativa

(1) Secretaria de Estado da Saúde
(2) Secretaria Municipal de Saúde
(3) Universidade pública
(4) Universidade privada
(5) Ministério da Saúde
(6) Consórcio intermunicipal
(7) Outros

** instituição que assume a responsabilidade técnica e administrativa em relação ao serviço

*** Essa pesquisa conta também com o apoio da Fundação de Amparo à Pesquisa do Estado de São Paulo -FAPESP**

© 2003 Equipe de Pesquisa Qualiaids. Todos os direitos reservados. É proibida a reprodução do conteúdo desta página em qualquer meio de comunicação, eletrônico ou impresso, sem autorização dos responsáveis pelo conteúdo.

2. Esse serviço de assistência à HIV/aids está localizado em uma unidade do tipo:

ASSINALE APENAS UMA ALTERNATIVA

(1) Ambulatório de assistência a várias especialidades
(2) Ambulatório de assistência especializada às pessoas vivendo com HIV/aids (ambulatório de DST/HIV/aids isolado)
(3) Ambulatório vinculado a Hospital
(4) Unidade básica de saúde

3. Quantos **pacientes** com HIV/aids **estão atualmente em seguimento** ambulatorial?

Considerar **todos** os pacientes em uso ou não de antiretroviral. Buscar informações no SICLOM e SISCEL

(1) Número de pacientes com idade acima de 13 anos ________
(2) Não é possível obter esses dados

4. Quantas **consultas médicas** são realizadas por esse serviço de HIV/aids por mês ?

✍ **Considere a média mensal de consultas realizadas por esse serviço no último ano*.**
✍ **Considere EXCLUSIVAMENTE o número de consultas médicas realizadas por infectologistas ou clínicos responsáveis pelo seguimento dos pacientes com HIV/aids. Não incluir consultas de ginecologia ou de outras especialidades**

(1) Consultas/mês de HIV/aids realizadas com pacientes acima de 13 anos: ______
(2) Não é possível obter esse dado.

* considere o período de 01 / Jan. a 31 / Dez. do ano anterior ao preenchimento do questionário

5. Quantos pacientes HIV/aids maiores de 13 anos **foram agendados** para consulta médica no último mês?

(1) Consultas agendadas de HIV/aids para maiores de 13 anos: ________

(2) Não é possível obter esse dado

6. Quantos pacientes HIV/aids maiores de 13 anos **faltaram** às consultas médicas agendadas no último mês?

(1) Número de faltas em consultas médicas no último mês em HIV/aids ______
(2) Não é possível obter esse dado

7. Quantos dias por semana o ambulatório de HIV/aids está aberto ao público?
(1) 1 dia por semana
(2) 2 dias por semana

(3) 3 dias por semana
(4) 4 dias por semana
(5) 5 dias por semana
(6) Mais de 5 dias por semana

8. Quantas horas por dia o ambulatório de HIV/aids está aberto ao público ?

(1) 4 horas por dia
(2) 6 horas por dia
(3) 8 horas por dia
(4) 12 horas por dia
(5) Mais de 12 horas por dia

9. Em relação aos estágios e/ou capacitações **oferecidos** por esse serviço pode-se dizer que:

Considerar como capacitação as atividades de educação permanente que visem o fortalecimento e/ou descentralização das ações. Não considerar palestras para a população geral ou grupos populacionais.

ASSINALE UMA OU MAIS ALTERNATIVAS

(1) Não se aplica, pois esse serviço não realiza nem oferece campo para estágios e capacitações
(2) São dirigidos a seus próprios alunos, porque esse serviço faz parte de uma instituição de ensino
(3) São dirigidos a alunos/profissionais de outros serviços ou instituições, com supervisão de profissionais "externos", vinculados a estas instituições
(4) São dirigidos a alunos/profissionais de outras instituições, com supervisão de profissionais desse serviço de HIV/aids

10. Qual o número de consultórios e salas para atendimento?

✍ Quando a sala for ocupada com mais de uma finalidade contabilizá-la apenas 1 vez

______ Consultórios médicos
______ Salas para atendimento de enfermagem
______ Salas para atendimento individual por outros profissionais
______ Salas para atendimento em grupo

11. Em relação ao acesso pode-se afirmar que:

ASSINALE UMA OU MAIS ALTERNATIVAS

(1) Esse serviço está localizado em região com fácil disponibilidade de transporte
(2) Esse serviço está localizado em região de difícil acesso a transportes coletivos
(3) O prédio tem planta física que facilita a circulação interna
(4) Existem barreiras físicas nesse serviço como escadas e outros que dificultam a circulação interna

12. A demanda de rotina do ambulatório é atendida:

ASSINALE APENAS UMA ALTERNATIVA

(1) Exclusivamente por médicos residentes ou estagiários
(2) A maior parte por médicos residentes
(3) A maior parte por médicos do próprio serviço
(4) Somente por médicos do próprio serviço

13. Quantos médicos responsáveis pelo seguimento ambulatorial de rotina a pacientes com HIV/aids existem nesse serviço?

✍ Não contar médicos residentes

(1) Número de médicos infectologistas atendendo HIV/aids:
(2) Número de médicos clínicos atendendo HIV/aids:

14. Em relação ao **tempo médio de experiência em HIV/aids** dos médicos infectologistas e clínicos-gerais responsáveis pelo seguimento ambulatorial de rotina a pacientes com HIV/aids, pode-se dizer que:

ASSINALE APENAS UMA ALTERNATIVA

(1) Todos os médicos possuem 5 ou mais anos de experiência
(2) Todos os médicos possuem menos de 5 anos de experiência
(3) A maioria dos médicos da equipe possui mais de 5 anos de experiência
(4) A maioria dos médicos da equipe possui menos de 5 anos de experiência
(5) Há pelo menos 1 médico com 5 ou mais anos de experiência

15. Preencha os campos abaixo indicando a **carga horária contratada semanal** dos médicos infectologistas e clínicos-gerais responsáveis pelo seguimento ambulatorial de rotina a pacientes com HIV/aids.

(1) Carga horária total/semanal de infectologistas que atendem HIV/aids: ________
(2) Carga horária total/semanal de clínicos-gerais que atendem HIV/aids: ________

16. Caracterize a disponibilidade dos profissionais responsáveis pelo atendimento ambulatorial de **ROTINA** dos pacientes com HIV/aids conforme quadro abaixo:

✍ na coluna A coloque o número de profissionais que atendem pacientes com HIV/aids por categoria
✍ enumere nas colunas B e C quantos profissionais são exclusivos para atendimento a pacientes com HIV/aids e quantos não o são.

**Áreas de especialidade**	**Número total de Profissionais**	**Número de profissionais que atendem HIV/aids segundo a exclusividade**	
	**A**	**B** N.º Prof. Exclusivos	**C** N.º Prof não exclusivos
(1) Enfermeiro			
(2) Psicólogo			

(3) Dentista			
(4) Farmacêutico			
(5) Assist. social			
(6) Auxiliar/técnico de enfermagem			

II. Sistema de registro e informações

17. Os prontuários dos pacientes com HIV/aids:

ASSINALE UMA OU MAIS ALTERNATIVAS

(1) São arquivados junto com os demais prontuários desse serviço (arquivo único)
(2) São arquivados separadamente (em arquivo exclusivo desses pacientes)
(3) São utilizados por todos os setores de assistência desse serviço (prontuário único)
(4) São utilizados apenas pelos profissionais da equipe que acompanha os pacientes de HIV/aids
(5) Outros

18. O registro do conteúdo da assistência prestada (história, condutas etc.) por profissional de nível universitário não-médico (psicólogo, assistente social, enfermeiro etc.) é realizado:

ASSINALE UMA OU MAIS ALTERNATIVAS

(1) No prontuário do paciente (prontuário único)
(2) Em livros ou fichas separadas
(3) Esse tipo de registro não é feito

19. Assinale as informações que possuem registro sistemático nesse serviço:

ATENÇÃO: os itens de 1 a 9 são informações sobre procedimentos/ações e a partir do item 10 são relacionadas a pacientes.

ASSINALE UMA OU MAIS ALTERNATIVAS

(1) Número de atendimentos e procedimentos
(2) Número de consultas por faixa etária
(3) Número de consultas por diagnóstico (infecções oportunistas/outras DSTs)
(4) Número de consultas por médicos
(5) Número de atendimentos em grupo
(6) Atendimentos a casos de exposição ocupacional
(7) Número total de faltas em consulta médica
(8) Número de faltas por paciente em consulta médica
(9) Número de exames laboratoriais realizados
(10)Número de pacientes por faixa etária
(11)Número de pacientes por sexo
(12)Número de pacientes soropositivos

(13) Número de pacientes com co-infecção por tuberculose<br>(14) Número de pacientes por diagnóstico (infecções oportunistas, outras DSTs)<br>(15) Número de pacientes em uso de anti-retroviral<br>(16) Primeiro atendimento no ano de cada paciente<br>(17) Número de óbitos<br>(18) Número de pacientes por esquema terapêutico com anti-retroviral<br>(19) Outros
20. No atendimento médico, o registro é feito:<br>**ASSINALE APENAS UMA ALTERNATIVA**<br>(1) Livremente em folhas em branco, a critério do profissional<br>(2) Em campos padronizados para preenchimento somente na 1ª consulta<br>(3) Em campos padronizados para preenchimento em todas as consultas
21. Em que outros atendimentos são utilizados formulários padronizados?<br>**ASSINALE UMA OU MAIS ALTERNATIVAS**<br>(1) No primeiro atendimento do paciente nesse serviço por profissional não-médico<br>(2) No atendimento de enfermagem<br>(3) No atendimento odontológico<br>(4) No atendimento com nutricionista<br>(5) No atendimento psicológico<br>(6) No atendimento em serviço social<br>(7) No atendimento em grupo<br>(8) No atendimento a casos de exposição ocupacional<br>(9) No atendimento de gestantes<br>(10) No atendimento de DST<br>(11) No atendimento de Tuberculose<br>(12) Outros<br>(13) Não são usados registros padronizados
22. De rotina, o preenchimento das fichas de notificação epidemiológica é realizado pelo seguinte profissional:<br>**ASSINALE UMA OU MAIS ALTERNATIVAS**<br>(1) Enfermeira<br>(2) Auxiliar/técnico de enfermagem<br>(3) Profissional administrativo<br>(4) Médico<br>(5) Outro profissional de nível universitário<br>(6) O médico preenche a parte clínica e outro profissional preenche o restante<br>(7) O enfermeiro preenche a parte clínica e outro profissional preenche o restante

(8) Não é realizado por esse serviço

III. Captação da demanda e primeiro atendimento de pacientes HIV/Aids

23. Quando o teste diagnóstico do HIV é oferecido nesse serviço, a orientação e o aconselhamento **pré-teste** são realizados:

ASSINALE UMA OU MAIS ALTERNATIVAS

(1) Em grupo por profissional de nível médio (auxiliar/técnico)
(2) Em grupo por profissional de nível universitário
(3) Individualmente por profissional de nível médio (auxiliar/técnico)
(4) Individualmente por profissional de nível universitário
(5) O aconselhamento não é realizado
(6) O teste não é oferecido nesse serviço

24. A orientação e o aconselhamento **pós-teste,** quando realizados nesse serviço**,** são feitos por quais profissionais?

ASSINALE UMA OU MAIS ALTERNATIVAS

(1) Profissional de nível médio (auxiliar/técnico)
(2) Profissional de nível universitário
(3) A orientação e o acompanhamento pós-teste não são realizados

25. Caso o paciente com sorologia positiva confirmada não retornar a esse serviço:

ASSINALE APENAS UMA ALTERNATIVA

(1) Aguarda o retorno espontâneo
(2) Convoca, tendo o cuidado de não quebrar o sigilo
(3) Convoca, apenas se for gestante, tendo o cuidado de não quebrar o sigilo
(4) Não se aplica, pois a testagem é anônima
(5) Não se aplica, pois esse serviço não oferece exame

26. Os pacientes que procuram atendimento pela primeira vez nesse serviço são **de rotina**:

ASSINALE APENAS UMA ALTERNATIVA

(1) Atendidos no mesmo dia por profissional não-médico de nível universitário
(2) Atendidos no mesmo dia por profissional de nível médio (auxiliar/técnico) da área de enfermagem e, se necessário, por consulta médica
(3) Atendidos no mesmo dia por profissional não-médico de nível universitário e, se necessário, por consulta médica
(4) Atendidos no mesmo dia por profissional não-médico de nível universitário e por consulta médica
(5) Atendidos em consulta médica no mesmo dia
(6) Agendados para outro dia para atendimento com profissional não-médico de nível universitário

(7) Agendados para outro dia para consulta médica<br>(8) Orientados a retornar nos dias subseqüentes para tentar vaga para consulta médica<br>(9) Outros

27. Nesse primeiro atendimento, as atividades realizadas são:

ASSINALE UMA OU MAIS ALTERNATIVAS

(1) Apresentação do serviço e de seu funcionamento
(2) Investigação sobre categoria de exposição
(3) Entrega de preservativos
(4) Triagem da área de moradia
(5) Avaliação de gravidade clínica
(6) Solicitação de exames
(7) Agendamento de consulta médica
(8) Orientação sobre formas de transmissão e métodos/práticas de prevenção
(9) Investigação e orientação sobre parceria sexual
(10) Investigação de tempo de diagnóstico
(11) Investigação sobre o uso anterior de anti-retroviral
(12) Outros

28. Quando o paciente de HIV/aids não é matriculado nesse serviço, na maioria das vezes ele é:

ASSINALE APENAS UMA ALTERNATIVA

(1) Encaminhado verbalmente para outro serviço
(2) Encaminhado com guia/carta para outro serviço
(3) Encaminhado com guia/carta para outro serviço após contato e/ou agendamento
(4) Não se aplica, pois todos os casos são absorvidos por esse serviço

29. Após o primeiro atendimento, o paciente sai desse serviço com a consulta de retorno agendada?

(1) Não
(2) Sim

30. Qual o intervalo **médio de tempo** entre o primeiro atendimento nesse serviço e a primeira consulta médica de HIV/aids (excluindo os casos graves)?

ASSINALE APENAS UMA ALTERNATIVA

(1) O primeiro atendimento sempre é a primeira consulta
(2) Menos de 1 semana
(3) De 1 a 4 semanas
(4) De 5 a 8 semanas
(5) Mais de 8 semanas

IV. Organização geral da assistência às pessoas que vivem com HIV/Aids
31. Quando chega a esse serviço, o paciente com consulta marcada com o médico:<br>**ASSINALE APENAS UMA ALTERNATIVA**<br>(1) Passa antes por atendimento com auxiliar/técnico de enfermagem<br>(2) Passa antes por atendimento com o enfermeiro<br>(3) Passa por atendimento prévio ao do médico com outro profissional de nível superior<br>(4) Passa em consulta médica sem nenhum outro atendimento prévio<br>(5) Passa por atendimento prévio ao do médico dependendo da necessidade do paciente<br>(6) Outros
32. Quando existe pré-consulta de rotina (atendimento realizado pela enfermagem, antes da consulta médica), sua finalidade é:<br>**ASSINALE UMA OU MAIS ALTERNATIVAS**<br>(1) Aferir peso e/ou pressão arterial<br>(2) Verificar queixas agudas e definir prioridades de atendimento<br>(3) Fornecer preservativos<br>(4) Verificar a adesão aos anti-retrovirais<br>(5) Outros<br>(6) Não se aplica, pois a pré-consulta não é realizada
33. São realizadas atividades com os pacientes na sala de espera?<br>**ASSINALE UMA OU MAIS ALTERNATIVAS**<br>(1) Habitualmente não<br>(2) Sim, através da discussão de temas propostos pelos pacientes<br>(3) Sim, habitualmente são passados vídeos educativos<br>(4) Sim, habitualmente são realizadas palestras<br>(5) Outros
34. As consultas médicas são agendadas:<br>**ASSINALE APENAS UMA ALTERNATIVA**<br>(1) Com hora marcada para cada paciente<br>(2) Por grupos de pacientes por hora (por bloco)<br>(3) Todos os pacientes no início do turno<br>(4) Para cada médico existe uma rotina<br>(5) As consultas não são agendadas antecipadamente

35. Quantas consultas são previstas por período de 4 horas de trabalho para **cada** infectologista e/ou clínico (agendadas e extras)?

ASSINALE APENAS UMA ALTERNATIVA

(1) Menos de 8 consultas
(2) 8 consultas
(3) 10 consultas
(4) 12 consultas
(5) 16 consultas
(6) 20 consultas
(7) acima de 20 consultas

36. O número de consultas médicas para **casos novos** é pré-estabelecido para cada médico por período?

(1) Não
(2) Sim

37. Nas consultas de **retorno**, o mais freqüente é que o paciente seja agendado:

ASSINALE APENAS UMA ALTERNATIVA

(1) Para o mesmo profissional
(2) Para o profissional com agenda disponível
(3) Com o médico responsável, mas para atendimento com diferentes estagiários (alunos ou residentes)
(4) O retorno não é agendado. O paciente é orientado a retornar em período estabelecido pelo médico ou pela rotina desse serviço
(5) Outros

38. Qual a forma de acesso ao atendimento **odontológico** nesse serviço?

ASSINALE UMA OU MAIS ALTERNATIVAS

(1) Agendamento conforme demanda do paciente
(2) Agendamento de avaliação periódica mesmo na ausência de queixas
(3) Pronto atendimento para queixas agudas
(4) Não há atendimento odontológico nesse serviço

39. Para as mulheres, qual a forma de acesso predominante ao atendimento médico **ginecológico** nesse serviço?

ASSINALE UMA OU MAIS ALTERNATIVAS

(1) Agendamento conforme demanda do paciente

(2) Agendamento de avaliação periódica mesmo na ausência de queixas

(3) Pronto atendimento para queixas agudas

(4) Não há rotina

(5) Não há ginecologista nesse serviço

40. Esse serviço possui padronizações de condutas (protocolos)?

ASSINALE UMA OU MAIS ALTERNATIVAS

(1) Sim, no primeiro atendimento nesse serviço
(2) Sim, na consulta médica inicial
(3) Sim, na consulta médica de retorno
(4) Sim, no atendimento por outros profissionais de nível universitário
(5) Sim, no atendimento a condições específicas (como co-infecção HIV/tuberculose, gestantes, acidentes de trabalho e outros)
(6) Outros
(7) Não há padronizações para as atividades da assistência

41. No caso de existirem padronizações, como foram elaboradas?

ASSINALE UMA OU MAIS ALTERNATIVAS

(1) Pelos profissionais desse serviço
(2) Foram adaptadas da experiência de outros serviços
(3) Por outras instâncias do Programa de DST/Aids
(4) Outros
(5) Não existem padronizações

V. Seguimento ambulatorial de rotina de pacientes HIV/Aids

42. Qual o **tempo médio** de duração da consulta do infectologista e/ou clínico para **consultas de casos novos ?**

ASSINALE APENAS UMA ALTERNATIVA

(1) 15 minutos
(2) 30 minutos
(3) 45 minutos
(4) 60 minutos

43. Qual o **tempo médio** de duração do atendimento do infectologista e/ou clínico para **consultas de retorno?**

ASSINALE APENAS UMA ALTERNATIVA

(1) 15 minutos
(2) 30 minutos
(3) 45 minutos
(4) 60 minutos

44. Durante a **consulta médica** de seguimento, **além dos procedimentos técnicos de rotina,** o médico:

ASSINALE UMA OU MAIS ALTERNATIVAS

(1) Preenche os formulários para dispensação de anti-retrovirais
(2) Investiga as dificuldades no uso de anti-retrovirais
(3) Orienta resumidamente a medicação
(4) Encaminha para outro profissional para orientação detalhada da medicação
(5) Orienta detalhadamente o uso de medicação
(6) Orienta o uso de preservativo
(7) Orienta o paciente sobre concepção/contracepção
(8) Orienta sobre exposição acidental entre pares discordantes
(9) Aborda questões relativas à sexualidade
(10) Agenda pessoalmente o retorno
(11) Faz outras orientações ou procedimentos
(12) Encaminha para outros atendimentos

45. Para os pacientes que **não estão em uso** de anti-retroviral, qual é o intervalo **médio** entre as consultas?

ASSINALE APENAS UMA ALTERNATIVA

(1) 30 dias
(2) 60 dias
(3) 90 dias

(4) 120 dias
(5) acima de 120 dias

46. Para os pacientes **em uso** de anti-retroviral, Qual é o intervalo **médio** entre as consultas de rotina?

ASSINALE APENAS UMA ALTERNATIVA

(1) 30 dias
(2) 60 dias
(3) 90 dias
(4) acima de 90 dias

47. Qual o intervalo de rotina para o retorno em consulta médica ou com profissional de nível superior quando é **introduzida** a terapia anti-retroviral nesse serviço?

ASSINALE APENAS UMA ALTERNATIVA

(1) 7 dias
(2) 15 dias
(3) 30 dias
(4) 60 dias
(5) Não existe uma rotina diferente no início da terapia anti-retroviral

48. Para os pacientes em seguimento, o uso correto da medicação geralmente é verificado através de:

ASSINALE UMA OU MAIS ALTERNATIVAS

(1) Questionamento sobre o uso correto
(2) Checagem de rotina em consulta médica sobre dificuldades com efeitos colaterais e posologia (horários e alimentação)
(3) Checagem de rotina sobre dificuldades relativas ao dia a dia (trabalho, fins de semana, festas etc.)
(4) Contagem dos medicamentos que sobraram
(5) Recordatório detalhado sobre as atividades diárias do paciente e o uso da medicação
(6) Checagem de anotações do paciente sobre o modo de uso diário, em caderno ou formulário específico
(7) Grupo de adesão
(8) Outros

49. Qual o mecanismo utilizado para garantir acesso aos anti-retrovirais quando o agendamento de rotina prevê um intervalo entre as consultas **de mais de 30 dias**?

ASSINALE APENAS UMA ALTERNATIVA

(1) Quando o paciente está estável leva prescrição para 60 dias
(2) A enfermeira ou o farmacêutico autoriza a retirada da medicação

(3) O paciente passa por consulta de enfermagem e, após orientações, retira a medicação previamente prescrita
(4) O paciente passa por atendimento com farmacêutico e, após orientações retira a medicação previamente prescrita
(5) O médico deixa prescrição pronta no serviço para o próximo mês
(6) O paciente sempre leva prescrição para 60 dias
(7) Isso não acontece

50. Quando um paciente apresenta dificuldades no tratamento com anti-retroviral:

ASSINALE UMA OU MAIS ALTERNATIVAS

(1) O retorno é realizado em intervalos menores
(2) O paciente é encaminhado para grupos de adesão
(3) O paciente é encaminhado para orientação individual com profissional de nível superior
(4) Há mudança de esquema terapêutico, se possível
(5) Há reforço da importância da adesão nas consultas médicas
(6) Outros

51. No caso do paciente ficar sem medicação, por razões imprevistas, ele é orientado a:

ASSINALE APENAS UMA ALTERNATIVA

(1) Agendar consulta médica
(2) Passar em consulta extra (encaixe na agenda ou pronto atendimento)
(3) Pegar receita com enfermagem ou farmacêutico até a próxima consulta
(4) Outros

52. A pós-consulta (atendimento de enfermagem após o atendimento médico) é realizada com a finalidade de:

ASSINALE UMA OU MAIS ALTERNATIVAS

(1) Agendar retorno
(2) Agendar exames
(3) Orientar uso de medicação
(4) Fornecer preservativos
(5) Orientar uso de preservativos
(6) Orientar encaminhamentos para outros serviços
(7) Orientar encaminhamentos para benefícios sociais
(8) Orientar sobre direitos civis
(9) Outros
(10) Não há pós-consulta

53. Em relação ao desejo de ter filhos por parte das pessoas que vivem com HIV/aids (homens e mulheres), os profissionais desse serviço:

ASSINALE UMA OU MAIS ALTERNATIVAS

(1) Não recomendam a concepção devido aos riscos para a saúde da mulher soropositiva
(2) Não recomendam a concepção devido ao risco de transmissão vertical
(3) Orientam sobre os riscos e discutem o melhor momento para a concepção
(4) Não recomendam a concepção, mas se a mulher engravidar, fazem o acompanhamento

54. Quando o paciente falta ao atendimento agendado, esse serviço:

ASSINALE UMA OU MAIS ALTERNATIVAS

(1) Aguarda retorno espontâneo em todos os casos
(2) Convoca paciente em uso de anti-retroviral
(3) Convoca os casos com maior gravidade clínica
(4) Convoca quando é caracterizado o abandono
(5) Convoca os casos que apresentam exames alterados
(6) Convoca todos os faltosos
(7) Outros
(8) Não se aplica, pois esse serviço não faz controle de faltosos.
(9) Não se aplica, pois esse serviço não trabalha com agendamento

55. Para os pacientes em uso de anti-retroviral, qual o critério de abandono utilizado por esse serviço?

ASSINALE UMA OU MAIS ALTERNATIVAS

(1) Duas faltas consecutivas na consulta médica (não importando o intervalo de tempo entre as consultas)
(2) 3 ou mais faltas consecutivas na consulta médica
(3) 2 meses de ausência no serviço
(4) De 3 a 5 meses de ausência no serviço
(5) 6 meses ou mais de ausência no serviço
(6) Após convocação sem comparecimento
(7) Outros
(8) Não há critério de abandono

56. Esse serviço faz controle do número de óbitos entre seus pacientes?

ASSINALE UMA OU MAIS ALTERNATIVAS

(1) Não de modo sistemático
(2) Sim, sempre que a família avisa
(3) Sim, através de informação proveniente do serviço de epidemiologia

(4)	Sim, esse serviço busca sistematicamente a informação em outros órgãos (por exemplo: veículos de comunicação, registros de óbitos do município, velórios e outros)

VI. Atendimento não agendado de pacientes HIV/Aids

57. De que maneira esse serviço soluciona o atendimento de pacientes “extras” ou não agendados?

ASSINALE UMA OU MAIS ALTERNATIVAS

(1) Há reserva de vagas na agenda dos médicos
(2) Os médicos do ambulatório fazem escala para atendimento desses pacientes
(3) Os pacientes são atendidos na dependência de vaga nas agendas médicas
(4) Esse serviço dispõe de médico(s) específico(s) para pronto atendimento a pacientes HIV/aids
(5) Os pacientes são encaminhados para atendimento em Pronto Socorro não específico para pacientes HIV/aids
(6) Os pacientes são sempre atendidos, independentemente da existência de vagas na agenda
(7) Outros

58. Qual a demanda mais freqüente para os atendimentos “extras” (consultas não agendadas para pacientes em seguimento)?

ASSINALE APENAS UMA ALTERNATIVA

(1) Intercorrências clínicas
(2) Término da medicação
(3) Falta à última consulta agendada
(4) Abandono de tratamento
(5) Resposta à convocação
(6) Fornecimento de atestado para benefícios sociais
(7) Não é possível obter essas informações
(8) O atendimento extra não é realizado
(9) Esse serviço não trabalha com agendamento prévio

59. Quais atividades são realizadas rotineiramente no atendimento **"extra" ou não agendado:**

ASSINALE UMA OU MAIS ALTERNATIVAS

(1) Avaliação de queixas clínicas
(2) Fornecimento de receita de anti-retroviral e outras medicações, quando necessário
(3) Avaliação e reorientação do uso de anti-retroviral e outras medicações
(4) Solicitação de exames
(5) Verificação da ocorrência de faltas anteriores
(6) Orientação sobre retorno para o atendimento de rotina
(7) Identificação das dificuldades em comparecer aos atendimentos agendados
(8) Fornecimento de atestado para benefício social
(9) Outros
(10) O atendimento "extra" não é realizado

VII. Características da assistência prestada pela equipe multiprofissional

60. Durante o atendimento, quais atividades são realizadas de rotina pelo **auxiliar/técnico de enfermagem**?

ASSINALE UMA OU MAIS ALTERNATIVAS

(1) Agendamento de exames e consultas
(2) Verificação de sinais vitais – peso, altura, temperatura, pressão arterial
(3) Verificação de queixas agudas que exijam providências
(4) Conferência do prontuário e do motivo do atendimento – resultados de exames etc.
(5) Orientação sobre o uso de preservativos
(6) Distribuição de preservativos
(7) Orientação para coleta de exames
(8) Orientação sobre o uso da medicação
(9) Discussão sobre as dificuldades de adesão
(10) Orientações nutricionais e dietéticas
(11) Grupos de adesão ou grupos sobre outros temas específicos
(12) Orientação sobre encaminhamentos para especialistas
(13) Coleta de sangue para exames laboratoriais
(14) Outros
(15) Esse serviço não dispõe desse profissional

61. Quais atividades são realizadas pelo **enfermeiro**?

ASSINALE UMA OU MAIS ALTERNATIVAS

(1) Recepção e acolhimento de casos novos
(2) Triagem de intercorrências
(3) Realização de grupos de adesão
(4) Realização de grupos sobre outros temas específicos
(5) Notificação epidemiológica
(6) Supervisão da equipe de enfermagem
(7) Visita domiciliar
(8) Outros
(9) Esse serviço não dispõe desse profissional

62. Na **consulta de enfermagem** (atendimento individual pelo enfermeiro) é realizado:

ASSINALE UMA OU MAIS ALTERNATIVAS

(1) Orientação sobre uso correto de medicação
(2) Discussão sobre adesão
(3) Orientação sobre as formas de transmissão e cuidados domésticos (biossegurança)
(4) Orientação sobre uso de preservativos
(5) Orientação sobre concepção / contraçepção
(6) Atendimento às DST (abordagem sindrômica)
(7) Atendimento aos casos de exposição ocupacional
(8) Outros
(9) O enfermeiro não realiza consulta de enfermagem
(10) Esse serviço não dispõe desse profissional

63. Quais atividades são realizadas pelo **psicólogo**?

ASSINALE UMA OU MAIS ALTERNATIVAS

(1) Recepção e acolhimento de casos novos
(2) Atendimento conjunto com médico e/ou serviço social
(3) Psicoterapia individual
(4) Psicoterapia de grupo
(5) Suporte para a equipe técnica desse serviço
(6) Triagem de intercorrências
(7) Grupos de adesão
(8) Grupos sobre outros temas específicos
(9) Visita domiciliar
(10) Atendimento e suporte para a família
(11) Outros
(12) Esse serviço não dispõe desse profissional

64. Quais atividades são realizadas pelo **assistente social**?

ASSINALE UMA OU MAIS ALTERNATIVAS

(1) Recepção e acolhimento de casos novos
(2) Discussão e apoio sobre estrutura familiar
(3) Discussão e orientação sobre questões trabalhistas
(4) Orientação sobre direitos civis e previdenciários
(5) Encaminhamentos para benefícios sociais (cesta básica, vale transporte etc.)
(6) Grupos de adesão
(7) Grupos sobre outros temas específicos
(8) Controle de faltosos e/ou busca ativa
(9) Atividades de reinserção social e/ou geração de renda

(10) Visita domiciliar
(11) Outros
(12) Esse serviço não dispõe desse profissional

65. As atribuições do **farmacêutico** são:

ASSINALE UMA OU MAIS ALTERNATIVAS

(1) Controle do suprimento e dispensação dos medicamentos
(2) Treinamento e supervisão dos auxiliares de farmácia (ou dos outros profissionais que auxiliam na farmácia)
(3) Dispensação de medicamentos
(4) Orientação individual dos pacientes quanto ao uso da medicação
(5) Supervisão e orientação da equipe médica sobre a adequação dos esquemas com anti-retroviral
(6) Controle de faltosos
(7) Grupos de adesão
(8) Outros
(9) Esse serviço não dispõe desse profissional

66. Como os diferentes profissionais da equipe atendem e encaminham os pacientes?

ASSINALE UMA OU MAIS ALTERNATIVAS

(1) O médico identifica as necessidades e encaminha para os diferentes profissionais (psicólogos, assistentes sociais e outros) ou para outras instituições
(2) Todos os profissionais encaminham para todos da equipe
(3) Os pacientes são obrigatoriamente atendidos, pelo menos uma vez, pelos diferentes profissionais da equipe básica (médico, psicólogo, assistente social e enfermeiro)
(4) Não existem outros profissionais com nível universitário além do médico

VIII. Medicamentos

67. Classifique os medicamentos abaixo com relação a sua disponibilidade nos últimos 6 meses:

NÃO DISPONÍVEL: não padronizado pelo município ou não adquirido há pelo menos 6 meses

NÃO UTILIZADO: não há demanda em razão do perfil clínico-imunológico dos pacientes

MARCAR UMA ALTERNATIVA POR MEDICAMENTO

Medicamentos	Não utilizado	Não disponível	Não faltou	Faltou: tempo médio de falta nos últimos 6 meses		
	A	B	C	D) Até 15 dias	E) 16 a 30 dias	F) Mais de 30 dias
(1) Azitromicina						
(2) Dapsona						

(3) Sulfadiazina						
(4) Sulfametoxazol-Trimetoprim						
(5) Cetoconazol						
(6) Fluconazol						
(7) Aciclovir comp.						
(8) Pirimetamina						

68. Nesse serviço a orientação do uso de anti-retroviral é realizada:

ASSINALE UMA OU MAIS ALTERNATIVAS

(1) Na consulta médica
(2) Por auxiliar/técnico de enfermagem
(3) Na farmácia, por farmacêutico ou pessoal auxiliar
(4) Na consulta do farmacêutico
(5) Na consulta do enfermeiro
(6) No atendimento individual por outros profissionais de nível superior
(7) Nos grupos de discussão sobre adesão

69. Nesse serviço, a dispensação de anti-retroviral na **primeira prescrição** é feita:

ASSINALE APENAS UMA ALTERNATIVA

(1) No mesmo dia
(2) Até 7 dias após a prescrição
(3) De 8 a 15 dias após a prescrição
(4) De 16 a 30 dias após a prescrição
(5) Acima de 30 dias após a prescrição
(6) Esse serviço não tem acesso a essa informação

70. O controle da dispensação de medicamentos é feito através de:

ASSINALE UMA OU MAIS ALTERNATIVAS

(1) Ficha de cadastro individual, com informações sobre esquema Terapêutico e data da retirada
(2) SICLOM (Sistema de Informação e Controle de Logística de Medicamento)
(3) Retenção da 2ª via da receita médica
(4) Fichas de prateleira para controle de estoque de medicamentos
(5) Livro de registro
(6) Planilha de controle de medicamentos
(7) Outros
(8) Esse serviço não tem esse tipo de controle

71. Ao realizar o controle de dispensação dos anti-retrovirais, este serviço retira os óbitos notificados, de forma a atualizar as informações sobre o número de pacientes em uso de anti-retrovirais?

(1) Não

(2) Sim

Não se aplica, pois esse serviço não dispensa anti-retroviral

72. Classifique os anti-retrovirais abaixo com relação a sua disponibilidade nos últimos 6 meses:

MARCAR UMA ALTERNATIVA POR MEDICAMENTO

Medicamentos	Não Disponível / Não utilizado	Não faltou	Faltou: Tempo médio de falta nos últimos 6 meses			
	A	B	C) Até 7 dias	D) 8 a 15 dias	E) 16 a 30 dias	F) mais de 30 dias
(1) Abacavir (ABV) comp. 300mg						
(2) Amprenavir (AMP) comp. 150mg						
(3) Atazanavir (ATZ) comp. 150mg						
(4) Atazanavir (ATZ) comp. 200mg						
(5) Didanosina (ddI) comp.100mg						
(6) Didanosina (ddI) comp.25mg						
(7) Didanosina EC comp.400mg						
(8) Didanosina EC comp.250mg						
(9) Efavirenz (EFV) caps. 600mg						
(10) Enfuvirtida (T20) fr.amp.90mg						
(11) Estavudina (D4T) comp 30 mg						
(12) Estavudina (D4T) comp 40mg						
(13) Indinavir (INV) comp. 400mg						
(14) Lamivudina (3TC) comp.150mg						
(15) Lopinavir/r (LPV) comp. 133mg						
(16) Nelfinavir (NFV) caps. 250mg						
(17) Nevirapina (NPV) caps. 200mg						
(18) Ritonavir (RTV) comp.100mg						
(19) Saquinavir (SQV) cps. duras 200mg						
(20) Saquinavir (SQV) cps. gelatinosas 200mg						
(21) Tenofovir (TFD) comp. 300mg						
(22) Zidovudina (AZT) comp. 100mg						
(23) Zidovudina (AZT) suspensão						

(24) Zidovudina (AZT) injetável						
(25) Zidovudina 300mg + Lamivudina 150mg						

( ) Esse serviço não possui essas informações

73. **Caso esse serviço não dispense** anti-retrovirais, a dispensação na unidade de referência:

ASSINALE UMA OU MAIS ALTERNATIVAS

(1) É demorada devido à falta freqüente de medicação
(2) É demorada devido às exigências burocráticas
(3) É demorada devido dificuldade de acesso (outra cidade, bairros afastados)
(4) A dispensação supre as necessidades desse serviço
(5) Não é possível informar porque não há integração com a unidade dispensadora.
(6) Não se aplica, pois esse serviço dispensa anti-retroviral

IX. Apoio diagnóstico e terapêutico

74. Com relação ao acesso às modalidades assistenciais dirigidas ao cuidado dos pacientes com HIV/aids, esse serviço dispõe:

(ESCOLHA UMA ALTERNATIVA POR LINHA)

Modalidade Assistencial	Disponível nesse serviço (A)	Disponível fora desse serviço		Não disponível (D)
		Com facilidade (B)	Com dificuldade (C)	
(1) CTA/COAS				
(2) ADT				
(3) HD- Hospital Dia				
(4) Internação convencional				
(5) Pronto Socorro (24horas)				

75. Quanto aos exames laboratoriais, esse serviço:

ASSINALE APENAS UMA ALTERNATIVA

(1) Coleta todos os exames
(2) Coleta parte dos exames no serviço e encaminha outros para coleta em laboratório de referência
(3) Encaminha todos os exames para coleta em laboratório de referência

76. Caracterize a disponibilidade de exames, assinalando o **tempo médio** de espera para o resultado

ESCOLHA UMA ALTERNATIVA POR EXAME

CONSIDERE O TEMPO A PARTIR DA SOLICITAÇÃO DO EXAME

✍ CONSIDERAR A DISPONIBILIDADE INDEPENDENTEMENTE DO EXAME SER REALIZADO DENTRO OU FORA DESSE SERVIÇO

EXAMES	Não disponível	**Disponível** com tempo médio de espera para o resultado de:					
	A)	B) até 7 dias	C) 8 a 15 dias	D) 16 a 30 dias	E) 31 a 45 dias	F) 46 dias ou mais	G) Imprevisível
(1) Amilase							
(2) Bilirrubina/frações							

(3) Colesterol							
(4) Creatinina							
(5) Glicose							
(6) TGO/TGP							
(7) Uréia							
(8) Triglicérides							
(9) Hemograma							
(10) Hemocultura Bactéria							
(11) Coprocultura							
(12) Urocultura							
(13) BAAR							
(14) PPD							
(15) Urina I/EAS (elementos anormais no sedimento urinário)							
(16) Parasitológico de fezes							
(17) Anti-HIV (Elisa)							
(18) Western-Blot							
(19) Imunofluor para HIV							
(20) S. Hepatite B							
(21) S. Hepatite C							
(22) S. Toxoplasmose							
(23) CD4/CD8							
(24) Carga Viral							

77. Faça a mesma caracterização em relação aos exames listados abaixo:

✍ **CONSIDERAR A DISPONIBILIDADE INDEPENDENTEMENTE DO EXAME SER REALIZADO DENTRO OU FORA DESSE SERVIÇO**

ESCOLHA UMA ALTERNATIVA POR EXAME

**OUTROS EXAMES**	**Não disponível**	**Disponível** com tempo médio de espera para o resultado de:					
	**(A)**	**B) Até 7 dias**	**C) 8 a 15 dias**	**D) 16 a 30 dias**	**E) 31 a 45 dias**	**F) 46 dias ou mais**	**G) Imprevisível**
(1) Rx Tórax							
(2) Rx Seios da Face							
(3) Rx Abdome							
(4) EDA							
(5) USG							
(6) Tomografia							
(7) Broncoscopia							

(8) Colposcopia							
(9) Anatomo Patológico							
(10) ECG							

78. A disponibilidade de cota para realização de exames de CD4 é, aproximadamente:

✍ **Multiplicar a cota mensal por 12 e dividir pelo número de pacientes**

(1) 1 exame por paciente por ano
(2) 2 exames por paciente por ano
(3) 3 exames por paciente por ano
(4) Mais de 3 exames por paciente por ano
(5) Exame não disponível

79. A disponibilidade de cota para realização de exames de carga viral é, aproximadamente:

✍ **Multiplicar a cota mensal por 12 e dividir pelo número de pacientes**

(1) 1 exame por paciente por ano
(2) 2 exames por paciente por ano
(3) 3 exames por paciente por ano
(4) Mais de 3 exames por paciente por ano
(5) Exame não disponível

80. Caracterize o acesso e o tempo médio de espera para atendimentos nas seguintes especialidades:

✍ **CONSIDERAR O ACESSO INDEPENDENTEMENTE DA ESPECIALIDADE SE CONCENTRAR DENTRO OU FORA DESSE SERVIÇO**

MARCAR UMA ALTERNATIVA POR LINHA

ESPECIALIDADES	Não disponível	Disponível com tempo médio de espera para acesso de:				
	A	B) até 15 dias	C) 16 a 45 dias	D) 46 a 60 dias	E) mais de 60 dias	F) imprevisível
(1) Ginecologia						
(2) Psiquiatria						
(3) Neurologia						
(4) Proctologia						
(5) Cardiologia						
(6) Oftalmologia						
(7) Cirurgia Geral						

(8) Odontologia						
(9) Psicologia						
(10) Serviço social						

X. Cuidados de Biossegurança

81. Em caso de acidente com material biológico a conduta é:

ASSINALE APENAS UMA ALTERNATIVA

(1) O funcionário é atendido no próprio serviço para orientações e conduta **até duas horas** após o acidente
(2) O funcionário é atendido em outro serviço para orientações e conduta **até duas horas** após o acidente
(3) O funcionário é atendido em outro serviço para orientações e conduta **após duas horas**
(4) Não existe fluxo estabelecido
(5) Informação não disponível

82. Os profissionais já receberam treinamento específico nos procedimentos a serem realizados nos casos de exposição ocupacional?

(1) Não
(2) Sim

83. O acondicionamento dos resíduos ou lixo contaminados é realizado:

ASSINALE UMA OU MAIS ALTERNATIVAS

(1) Por profissionais desse serviço treinados para esse fim
(2) Por profissionais desse serviço treinados, com supervisão da enfermeira
(3) Por profissionais desse serviço não treinados até o momento
(4) Por profissionais terceirizados treinados com supervisão
(5) Por profissionais terceirizados treinados
(6) Por profissionais terceirizados não treinados até o momento
(7) Outros

XI. Capacitação de recursos humanos

84. No último ano, esse serviço promoveu ou enviou quais profissionais para reciclagem/capacitação?

ASSINALE UMA OU MAIS ALTERNATIVAS

(1) Médicos
(2) Enfermeiros
(3) Assistentes sociais
(4) Psicólogos

(5) Nutricionistas
(6) Dentistas
(7) Farmacêuticos
(8) Auxiliares/técnicos de enfermagem
(9) Auxiliares/técnicos em odontologia
(10) Pessoal administrativo
(11) Auxiliares de serviços gerais (limpeza)
(12) Para nenhuma categoria
(13) Outros

85. No último ano, esse serviço ofereceu suporte financeiro para seus profissionais participarem de treinamentos, congressos e similares?

(1) Não
(2) Sim

86. Nesse serviço existe material de consulta, como textos técnicos e similares?

ASSINALE APENAS UMA ALTERNATIVA

(1) Não
(2) Sim, mas não existe espaço físico reservado para a consulta
(3) Sim. O material está disponível em sala de leitura ou equivalente

87. Esse serviço promove sistematicamente reuniões para discussão de casos e condutas clínicas?

ASSINALE APENAS UMA ALTERNATIVA

(1) Não
(2) Sim, semanalmente
(3) Sim, quinzenalmente
(4) Sim, mensalmente
(5) Sim, apenas quando há necessidade

XII. Gerência

88. A responsabilidade **administrativa** (controle de faltas, férias, alocação de pessoal, contratação) sobre a equipe de assistência à aids nesse serviço é exercida:

ASSINALE APENAS UMA ALTERNATIVA

(1) Pelo gerente desse serviço, que é um profissional não vinculado ao Programa de DST/Aids
(2) Pelo gerente desse serviço, que é um profissional que integra o Programa de DST/Aids
(3) Diretamente pela coordenação municipal do Programa de DST/Aids

(4) Diretamente pela Secretaria Municipal de Saúde
(5) Outros

89. O gerente administrativo especificado anteriormente possui formação para a atividade gerencial?

ASSINALE APENAS UMA ALTERNATIVA

(1) Não
(2) Sim, através de especialização em saúde pública
(3) Sim, através de especialização em administração em saúde
(4) Sim, através de outros cursos de capacitação na área gerencial
(5) Outros

90. A coordenação **técnica** (responsável pela assistência, reuniões, supervisão etc.) imediata das atividades ambulatoriais desse serviço de HIV/aids é realizada:

ASSINALE APENAS UMA ALTERNATIVA

(1) Pelo gerente desse serviço especializado em HIV/aids
(2) Por um coordenador ou chefia técnica da equipe de assistência ambulatorial **(mesmo que não formalizado)**
(3) Pelo coordenador municipal de DST/aids
(4) Por um profissional que acumula a função de coordenação local e municipal do Programa de DST/Aids
(5) Não existe coordenação direta das atividades de assistência ambulatorial

91. As atividades desenvolvidas **nesse serviço** pelo coordenador técnico da assistência ambulatorial são:

ASSINALE UMA OU MAIS ALTERNATIVAS

(1) Exclusivamente gerenciais
(2) A maior parte gerencial e uma pequena parte assistencial
(3) Uma grande parte assistencial e pequena parte gerencial
(4) Gerência e assistência são realizadas ao mesmo tempo ou na mesma proporção
(5) Atividades de ensino
(6) Outras
(7) Não se aplica, pois não há responsável técnico

92. O coordenador técnico especificado na questão anterior exerce atividades de coordenação técnica em serviços HIV/aids há:

(1) Menos de 1 ano
(2) Entre 1 e 3 anos
(3) Entre 3 e 6 anos
(4) Mais de 6 anos
(5) Não há responsável técnico

93. Esse serviço realiza reuniões de trabalho:

ASSINALE APENAS UMA ALTERNATIVA

(1) Semanalmente
(2) Quinzenalmente
(3) Mensalmente
(4) Sem periodicidade definida
(5) Nunca realiza

94. Que tipo de reuniões de trabalho esse serviço realiza **periodicamente**?

ASSINALE UMA OU MAIS ALTERNATIVAS

(1) Reuniões de caráter gerencial (administrativas)
(2) Reuniões técnicas/administrativas com toda a equipe multiprofissional
(3) Reuniões técnicas por categoria profissional
(4) Reuniões ampliadas com a participação de pacientes ou de entidades representativas (ONGs)
(5) Outras
(6) Não há reuniões

95. Quais membros da equipe participam das **reuniões multiprofissionais**:

ASSINALE UMA OU MAIS ALTERNATIVAS

(1) Diretor técnico
(2) Diretor administrativo
(3) Chefia médica
(4) Médicos
(5) Chefia de enfermagem
(6) Enfermeiros
(7) Assistente social
(8) Psicólogos
(9) Farmacêuticos
(10) Auxiliares/técnicos de enfermagem
(11) Não há esse tipo de reunião

96. Que tipo de atividades esse serviço oferece para minimizar o sofrimento dos profissionais que atendem os pacientes de HIV/aids?

ASSINALE UMA OU MAIS ALTERNATIVAS

(1) Grupos terapêuticos
(2) Atendimento individual com psicólogos e/ou psiquiatras
(3) Atividades de lazer e integração (coral, festas, caminhadas etc.)
(4) Atividades de relaxamento (ioga, shiatsu, tai-chi-chuan, massagens etc.)

(5) Outras
(6) Não existem essas atividades

97. Quanto ao suporte técnico-administrativo aos profissionais, são realizadas:

ASSINALE UMA OU MAIS ALTERNATIVAS

(1) Supervisões técnicas
(2) Supervisões administrativas
(3) Supervisão institucional
(4) Supervisão clínica para os psicólogos
(5) Não há nenhum tipo de supervisão

98. Esse serviço utiliza os dados epidemiológicos disponíveis da região para o planejamento de suas atividades como, por exemplo, o número e o perfil dos casos notificados?

ASSINALE APENAS UMA ALTERNATIVA

(1) Não
(2) Não, pois os dados não estão disponíveis
(3) Não, pois os dados estão desatualizados
(4) Sim

99. No último ano, esse serviço realizou o planejamento de suas atividades:

ASSINALE UMA OU MAIS ALTERNATIVAS

(1) Através da continuidade das atividades desenvolvidas no ano anterior
(2) Através do cumprimento das metas estabelecidas pela equipe de planejamento do nível central
(3) Através de plano elaborado pela equipe local desse serviço
(4) Conforme definido pelo diretor técnico
(5) Conforme articulação da gerência local com a Coordenação de DST/Aids
(6) Através de plano elaborado pela equipe e por representantes de pacientes
(7) Outros
(8) Não houve planejamento local

100. De que modo o serviço participa da priorização dos recursos financeiros destinados ao financiamento das ações de hiv/aids?

ASSINALE APENAS UMA ALTERNATIVA

(1) O gerente reune-se com a equipe e com instâncias superiores
(2) O gerente reune-se apenas com as instâncias superiores
(3) Não há participação direta desse serviço nas decisões
(4) O gerente reune-se com a equipe, com usuários (comissões municipais, fóruns ou similares) e instâncias superiores

101. Nos últimos 6 meses, esse serviço teve dificuldades gerenciais com:

ASSINALE UMA OU MAIS ALTERNATIVAS

(1) Acesso a exames de laboratório em geral
(2) Acesso a exames de CD4/Carga Viral
(3) Medicação anti-retroviral
(4) Medicação contra infecções oportunistas
(5) Encaminhamentos para especialidades
(6) Vagas para internação
(7) Vagas em hospital dia
(8) Vagas em maternidade
(9) Vaga para assistência domiciliar terapêutica
(10) Contratação de recursos humanos de nível superior
(11) Contratação de recursos humanos de nível médio (auxiliar/técnico)
(12) Aquisição de material de consumo
(13) Aquisição de material permanente
(14) Outros

102. Diante das dificuldades enfrentadas no gerenciamento, quais as medidas tomadas na busca de soluções?

ASSINALE UMA OU MAIS ALTERNATIVAS

(1) Encaminhamento dos problemas para a direção local
(2) Notificação à Coordenação Municipal do Programa DST/Aids
(3) Notificação à Coordenação Estadual de DST/Aids
(4) Notificação à Coordenação Nacional de DST/Aids
(5) Comunicado às instâncias de controle social (fórum de ONGs, Conselho Municipal de Aids, Conselho Municipal de Saúde)
(6) Contato com outros serviços ou instâncias gerenciais para remanejamento de insumos
(7) Reunião com pacientes
(8) Aguardo de providências de outras instâncias gerenciais
(9) Aquisição de insumos com verba de pronto pagamento ou com recursos de projetos
(10) Outros

103. A avaliação das atividades assistenciais é:

ASSINALE APENAS UMA ALTERNATIVA

(1) Realizada periodicamente para reprogramação das atividades
(2) Realizada esporadicamente, quando necessária
(3) Realizada anualmente para prestação de contas
(4) Realizada pela coordenação estadual ou nacional do programa

(5) Não é realizada

104. As avaliações já realizadas nesse serviço basearam-se em:

ASSINALE UMA OU MAIS ALTERNATIVAS

(1) Dados de produção ambulatorial
(2) Dados epidemiológicos
(3) Relatórios de atividades
(4) Levantamento da satisfação dos usuários
(5) Auditoria de prontuários
(6) Levantamento do perfil da demanda
(7) Não foram realizadas avaliações
(8) Outros

105. Há alguma forma organizada para encaminhar as reclamações dos pacientes?

ASSINALE UMA OU MAIS ALTERNATIVAS

(1) Não
(2) Caixa ou livro de sugestões e reclamações
(3) Linha telefônica municipal específica para reclamações e sugestões
(4) Sistema de ouvidoria (desse serviço, da secretaria etc.)
(5) Diretamente junto à direção local desse serviço ou ao conselho gestor
(6) Outros

106. Existe participação organizada dos pacientes no encaminhamento de soluções de problemas?

ASSINALE UMA OU MAIS ALTERNATIVAS

(1) Não
(2) Sim, através de ONGs
(3) Sim, através de conselho gestor ou similar
(4) Sim, através de outras formas de organização

107. Existe integração rotineira desse serviço com outros órgãos ou instituições não diretamente vinculadas à saúde?

ASSINALE UMA OU MAIS ALTERNATIVAS

(1) Não
(2) Sim, com o setor judiciário
(3) Sim, com ONGs
(4) Sim, com outras secretarias do município ou do estado, como a da educação e a do serviço social
(5) Sim, com igrejas ou entidades filantrópicas
(6) Sim, com outros órgãos
